# Comparison between angiography or intravascular imaging guided coronary rotational atherectomy

**DOI:** 10.1186/s12872-026-06411-6

**Published:** 2026-08-11

**Authors:** Yichen Liu, Xiaofei Gao, Jingyu Zhao, Ling Lin, Zhicheng Li, Jiaxian Song, Shouming Zhao, Xiangquan Kong

**Affiliations:** 1 Department of Cardiology, The First People’s Hospital of Taicang, No. 58 Changsheng South Road, Chengxiang Town, Taicang City, Jiangsu Province 215400 China; 2 Department of Cardiology, Nanjing First Hospital, Nanjing Medical University, No. 68 Changle Road, Nanjing, Jiangsu Province 210006 China; 3 Department of Cardiology, Huainan Xinhua Medical Group Xinhua Hospital, Huainan, Anhui Province China; 4 Department of Cardiology, Zhangjiagang Hospital Affiliated to Soochow University, The First People’s Hospital of Zhangjiagang City, Zhangjiagang, Jiangsu Province China

**Keywords:** Intravascular imaging, Rotational atherectomy, Coronary angiography, Major adverse cardiac events, Propensity score matching

## Abstract

**Background:**

Rotational atherectomy (RA) is a cornerstone technique for the management of severely calcified coronary lesions during percutaneous coronary interventions. Although intravascular imaging (IVI) provides superior anatomical guidance, its long-term prognostic impact in RA remains underexplored.

**Objectives:**

This study aimed to compare 12-month clinical outcomes between IVI-guided and coronary angiography (CAG)-guided RA.

**Methods:**

This retrospective analysis included 468 patients who underwent RA. Following 1:1 propensity score matching（PSM）, 306 patients (153 per group) were compared: IVI-guided versus angiography (CAG)-guided RA. The primary endpoint was 12-month major adverse cardiac events (MACE). A stratified Cox proportional hazards model, incorporating variables selected via least absolute shrinkage and selection operator (LASSO) regression, was employed for the primary adjusted analysis to mitigate overfitting.

**Results:**

Compared with CAG guidance, IVI-guided RA was associated with the use of larger burrs and stent diameters, as well as greater contrast volume (all *P*<0.05). At 12 months, IVI-guided RA was associated with significantly lower rates of MACE (7.2% vs. 15.0%; *P* = 0.033), cardiac death (2.0% vs. 8.5%; *P* = 0.020), and all-cause mortality (2.6% vs. 11.8%; *P*<0.005). In the stratified multivariable Cox model adjusting for preoperative renal function, IVI guidance remained independently associated with a lower hazard of MACE (adjusted hazard ratio [HR] 0.42, 95% confidence interval [CI] 0.20–0.86; *P* = 0.018).

**Conclusions:**

In this observational study, IVI-guided RA was associated with reduced 12-month MACE and all-cause mortality compared to CAG-guided RA. These findings generate hypotheses regarding the potential role of IVI guidance in this high-risk setting and underscore the need for validation in prospective, randomised trials.

**Supplementary Information:**

The online version contains supplementary material available at https://doi.org/10.1186/s12872-026-06411-6.

## Introduction

Rotational atherectomy (RA) is a pivotal strategy for the management of severely calcified coronary lesions, particularly when conventional balloons fail to cross or adequately expand a lesion [1]. By modifying calcific plaques, RA facilitates improved vessel preparation and enables optimal stent deployment.

Intravascular imaging (IVI), including intravascular ultrasound (IVUS) and optical coherence tomography (OCT), provides high-resolution visualisation of coronary anatomy and plaque morphology, which is critical for guiding procedural decisions and device selection [2, 3]. Numerous clinical trials comparing long-term clinical outcomes between IVI-guided and coronary angiography (CAG)-guided percutaneous coronary intervention (PCI) showed significant clinical benefits with IVI [4–6].

Despite the established value of IVI in complex PCI, large-scale studies comparing the long-term prognosis of IVI-guided versus conventional CAG-guided approaches in patients specifically undergoing RA remain scarce. Accordingly, this study compared 12-month clinical outcomes between IVI-guided and CAG-guided RA with the aim of contributing to the evidence base for treatment strategies in this population.

## Methods

### Study design and population

**Fig. 1:**
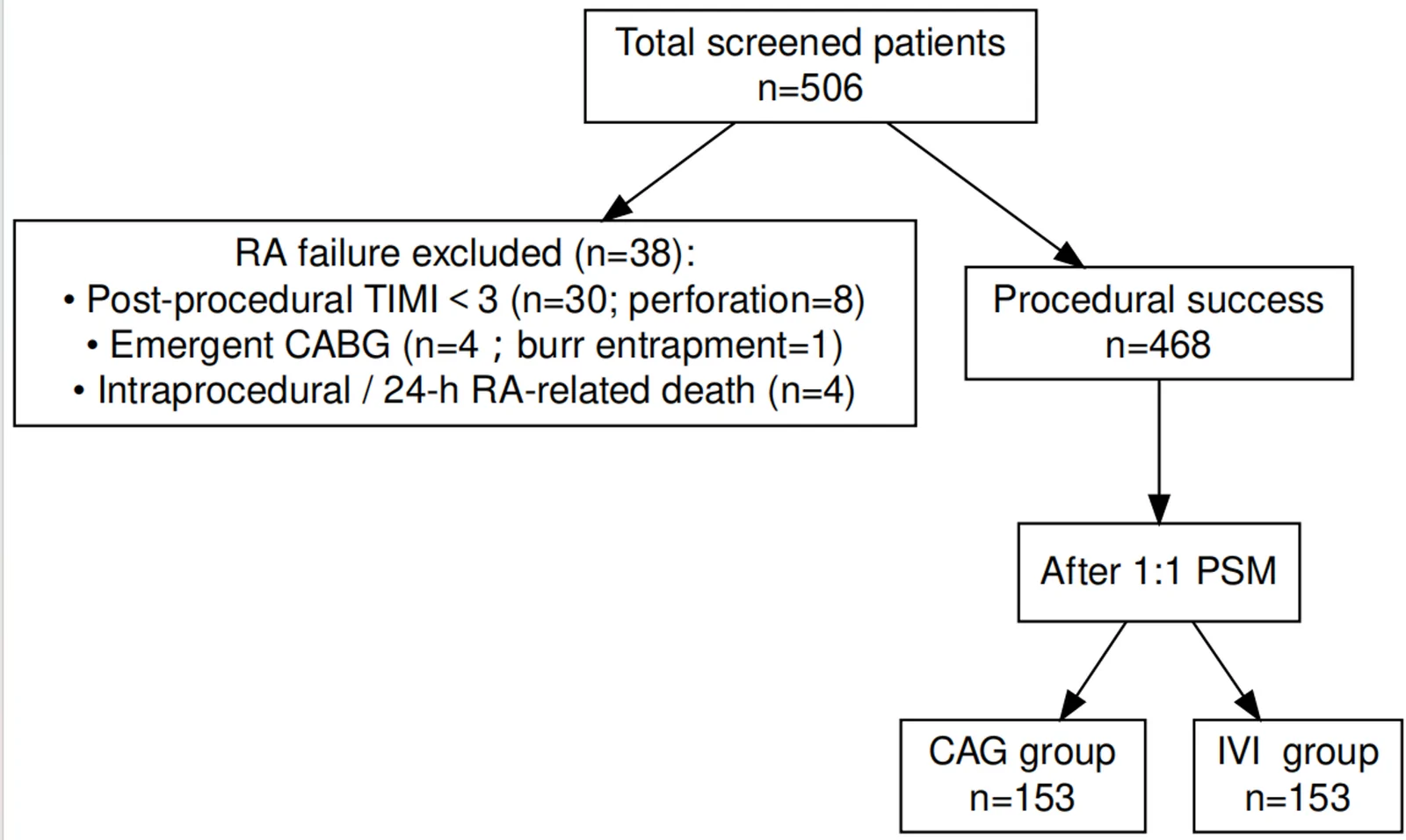
STROBE flow diagram of patient screening, exclusion and PSM: A total of 506 consecutive patients who received rotational atherectomy (RA) were initially screened. Thirty-eight patients were excluded due to procedural RA failure, including coronary perforation, burr entrapment, emergent coronary artery bypass grafting (CABG), and intraprocedural or 24-hour RA-associated death. Ultimately, 468 patients with technically successful RA were enrolled. A 1:1 greedy nearest-neighbor PSM analysis with a caliper width of 0.1 standard deviation (SD) was conducted to balance baseline confounders, resulting in 153 patients within the coronary angiography (CAG)-guided cohort and another 153 patients in the intravascular imaging (IVI)-guided cohort. Standardized mean differences (SMD) were calculated to evaluate covariate balance post-matching. Abbreviations: CABG, coronary artery bypass grafting; CAG, coronary angiography; IVI, intravascular imaging; PSM, propensity score matching; SMD, standardized mean difference

In this retrospective, multicentre cohort study, we screened 506 patients who underwent RA between January 2015 and August 2024 at participating cardiac centres in China. The patient screening, exclusion, and matching process is illustrated in the STROBE-compliant flowchart (Fig. 1). Patients were stratified into two cohorts based on intraprocedural imaging guidance: an IVI-guided group (IVUS or OCT) and a CAG-guided group. All participating institutions adhered to standardised RA protocols, ensuring uniformity in procedural execution and perioperative management [7, 8]. To ensure that the comparison of long-term outcomes reflected the efficacy of the imaging guidance strategy itself, rather than differences in procedural feasibility, we excluded patients who experienced procedural failure during RA (e.g., due to burr entrapment, coronary perforation, or failure to achieve procedural success criteria) prior to PSM. This approach minimized confounding by differential procedural success.

### Inclusion and exclusion criteria

Inclusion criteria were: (1) age ≥ 18 years; and (2) presence of severe coronary artery calcification in at least one vessel that was successfully treated with RA. In alignment with established guidelines, severe calcification was defined fluoroscopically as radio-opacities along the vessel wall without cardiac motion before contrast injection or, on IVI, as ≥ 270° of high-density echoes with associated acoustic shadowing in a cross-sectional view [7, 9, 10].

Exclusion criteria were: (1) RA failure: (a) failure to meet the procedural success criteria; (b) experience of severe RA-related complications, including coronary perforation, burr entrapment, or an ACC/AHA type C–F dissection necessitating procedure termination; (c) requirement for emergent coronary artery bypass grafting during the index procedure; or (d) occurrence of intraprocedural or 24-hour RA-associated cardiac death. (2) absence of follow-up data.

### Procedures and definitions of success

All rotational atherectomy (RA) procedures were performed according to established guidelines. After successful crossing of the target lesion using a dedicated RA guidewire (such as Rotawire), the initial burr size was selected based on preprocedural CAG or IVI assessment. Ablation was performed using a “pecking motion” technique at 130,000–220,000 rpm, with deceleration limited to 5,000 rpm. Each run was restricted to < 20 s, followed by irrigation with heparinized saline (with vasodilators if needed) to minimise slow-flow/no-reflow risk and facilitate burr cooling [7, 9, 10]. Decisions to upsize the burr, adjunctive balloon angioplasty, or drug-eluting stent (DES) implantation were operator-dependent.

Procedural success was defined as: (1) residual stenosis of the target vessel ≤ 30% in orthogonal views; and (2) restoration of Thrombolysis in Myocardial Infarction (TIMI) Grade III flow [7]. All intraoperative complications were managed. Post-procedure, all patients received dual antiplatelet therapy (DAPT) and guideline-directed secondary prevention. Pre- and post-operative imaging data were systematically archived.

### Clinical endpoints and follow-up

The primary adverse clinical endpoint was major adverse cardiac events (MACE), a composite of cardiac death, target vessel myocardial infarction (TV-MI), and clinically driven target lesion revascularisation (TLR). TV-MI was defined as an elevation in cardiac troponin above the 99th percentile upper reference, accompanied by ischemic symptoms or electrocardiographic changes. Secondary endpoints included the individual components of MACE, all-cause death, clinically driven target-vessel revascularisation (TVR), and a composite endpoint of all acute myocardial infarction (AMI) events. Given the anticipated low event rates for individual MI subtypes, this composite AMI endpoint was prespecified to ensure adequate statistical power for between-group comparisons. It encompassed target-vessel, periprocedural, and non–target-vessel MI, as defined by the Fourth Universal Definition.

Follow-up was conducted at 1, 3, 6, and 12 months post-procedure via outpatient visits, structured telephone interviews, and electronic medical record review. The endpoint was the first MACE event or completion of 12 months of follow-up; patients lost to follow-up were censored at their last confirmed contact date.

### Propensity Score Matching (PSM)

Two separate 1:1 greedy nearest-neighbor PSM analyses (caliper width = 0.1 SD) were performed to eliminate baseline confounding:

1. Primary PSM cohort: Matching covariates included baseline variables with *P* < 0.05 (bifurcation lesions, chronic total occlusion [CTO]) and established outcome predictors (estimated glomerular filtration rate [eGFR] [1], diabetes mellitus [11], left ventricular ejection fraction [LVEF] [12]). Baseline characteristics are presented in Table 1; standardized mean differences (SMD) for covariate balance are listed in *Supplementary Table S1*,with an absolute SMD < 0.1 considered indicative of adequate balance.
2. Stringent expanded PSM (sensitivity cohort): All demographic, clinical and angiographic covariates were incorporated into matching, generating 147 paired patients. Complete SMD values are shown in *Supplementary Table S2*.

**Table 1:** Baseline before and after PSM [n (%)]

Characteristics	Before PSM			PSM			
	IVI (*n* = 305)	CAG (*n* = 163)	*P*	IVI (*n* = 153)	CAG (*n* = 153)		*P*
Age, y	71.59 ± 8.18	72.69 ± 8.91	0.139	71.65 ± 8.38		72.67 ± 8.40	0.373
Male	193 (63.3%)	98 (60.1%)	0.502	100 (65.4%)		93 (60.8%)	0.407
Hypertension	238 (78.0%)	124(76.1%)	0.630	119 (77.8%)		117 (76.5%)	0.785
Diabetes mellitus	117 (38.4%)	63 (38.7%)	0.951	49 (32.0%)		59 (38.6%)	0.232
Dyslipidemia	177 (58.0%)	105(64.4%)	0.252	80 (52.3%)		96 (62.7%)	0.064
Previous Renal Insufficiency	59 (19.3%)	38 (23.3%)	0.313	26 (17.0%)		31 (20.3%)	0.463
Previous dialysis	7 (2.3%)	5 (3.1%)	0.760	2 (1.3%)		5 (3.3%)	0.448
Previous myocardial infarction	29 (9.5%)	18 (11.0%)	0.599	12 (7.8%)		15 (9.8%)	0.545
Previous PCI	79 (25.9%)	44 (27.0%)	0.798	39 (25.5%)		42 (27.5%)	0.697
Previous CABG	4 (1.3%)	1 (0.6%)	0.662	1 (0.7%)		0 (0.0%)	> 0.999
Previous peripheral vascular disease	19 (6.2%)	16 (9.8%)	0.160	12 (7.8%)		16 (10.5%)	0.428
Current smoking	65 (21.3%)	38 (23.3%)	0.619	37 (24.2%)		35 (22.9%)	0.788
Clinical syndrome			0.731				0.515
ACS	260 (85.2%)	137(84.0%)		133 (86.9%)		129 (84.3%)	
Stable angina pectoris	45 (14.8%)	26 (16.0%)		20 (13.1%)		24 (15.7%)	
Preoperative LVEF, %	58.54 ± 10.06	57.86 ± 10.35	0.220	58.63 ± 9.85		58.70 ± 9.51	0.609
Preoperative eGFR, ml/(min·1.73 m²)	77.47 ± 23.11	74.87 ± 24.24	0.140	78.48 ± 21.00		76.40 ± 23.30	0.467
Procedure period (3 categories)			0.028				0.149
2015–2018	87(29%)	66 (41%)		46(30%)		62 (41%)	
2019–2021	101(33.%)	48(29.4%)		50(32.7%)		45(29%)	
2022–2024	117(38%)	49(30%)		57(37%)		46(30%)	
*Angiographic Baseline Data*							
Multivessel lesions	276 (90.5%)	151(92.6%)	0.434	137 (89.5%)		141 (92.2%)	0.428
Bifurcation lesions	129 (42.3%)	51 (31.3%)	0.020*	46 (30.1%)		45 (29.4%)	0.900
CTO lesions	38 (12.5%)	38 (23.3%)	0.002*	29 (19.0%)		28 (18.3%)	0.883
Target vessel							
LM	17 (5.6%)	9 (5.5%)	0.981	7 (4.6%)		8 (5.2%)	0.791
LAD	236 (77.4%)	121(74.2%)	0.446	114 (74.5%)		115 (75.2%)	0.895
LCX	19 (6.2%)	8 (4.9%)	0.559	9 (5.9%)		8 (5.2%)	0.803
RCA	55 (18.0%)	34 (20.9%)	0.458	31 (20.3%)		30 (19.6%)	0.886

Continuous variables are expressed as mean ± standard deviation (SD), and categorical variables are expressed as number (percentage) [n(%)]. Statistical comparisons were performed using the Wilcoxon rank-sum test for continuous variables and the χ² test (or Fisher’s exact test for variables with small expected frequencies, e.g., previous CABG history) for categorical variables

*Abbreviations*: *ACS* Acute coronary syndrome, *LVEF* Left ventricular ejection fraction, *eGFR* Estimated glomerular filtration rate, *PCI* Percutaneous coronary intervention, *CABG* Coronary artery bypass grafting, *CTO* Chronic total occlusion, *LM* Left main coronary artery, *LAD* Left anterior descending artery, *LCX* Left circumflex artery, *RCA* Right coronary artery

*P < 0.05 indicates a statistically significant difference

### Statistical analysis

Continuous variables are presented as mean ± standard deviation and compared using the Wilcoxon rank-sum test. Categorical variables are reported as number (percentage) and compared using the χ² test or Fisher’s exact test, as appropriate.

#### Variable selection and model building

In the matched cohort (34 MACE events), we used least absolute shrinkage and selection operator (LASSO) regression with 10-fold cross-validation to select variables for a parsimonious Cox model, adhering to the rule of thumb of ≥ 10 events per variable. The treatment assignment (IVI- vs. CAG-guided) was prespecified for inclusion.

#### Model specification

Because the proportional hazards assumption was violated for the procedural period (global test of Schoenfeld residuals *p* < 0.05), the final multivariable Cox model was stratified by this variable. The model included the treatment group and the covariate selected by LASSO (preoperative eGFR < 60 mL/(min·1.73 m²)).

#### Survival analysis

Time-to-event outcomes were analysed using Kaplan-Meier curves, with between-group differences assessed by the log-rank test.

#### Sensitivity analyses

To evaluate the robustness of our primary study findings, we performed four pre-specified sensitivity analyses: (1) replication of the primary multivariable Cox regression model within an expanded, fully adjusted PSM cohort; (2) assessment of temporal confounding via cumulative hazard curves stratified by procedural periods; (3) comparison of rotational atherectomy procedural success rates across different guidance strategies and intravascular imaging subtypes to rule out selection bias; (4) validation of covariate balance and 12-month MACE survival profiles for the stringent sensitivity PSM cohort. Full methodological details and complete outputs of all sensitivity analyses are presented in the Supplementary Materials.

All statistical tests were two-sided, with a *P* < 0.05 considered statistically significant. All analyses were performed using R software (version 4.2.1).

## Results

### Baseline and procedural characteristics

Before PSM, 468 patients (IVI: 305, CAG: 163) were included. The IVI group had more bifurcation lesions (42.3% vs. 31.3%; *P* = 0.020) and fewer CTOs (12.5% vs. 23.3%; *P* = 0.002). Procedurally, IVI-guided cases utilised larger burrs, larger stents, greater contrast volume, and longer operation times (all *P* < 0.05; Tables 1 and 2).

**Table 2:** Procedural characteristics before and after PSM [n (%)]

Characteristics	Before PSM			PSM		
	IVI *n* = 305	CAG *n* = 163	*P*	IVI *n* = 153	CAG *n* = 153	*P*
Vascular access			0.078			0.212
Single arterial access	281 (92.1%)	157 (96.3%)		142 (92.8%)	147 (96.1%)	
Dual arterial access	24 (7.9%)	6 (3.7%)		11 (7.2%)	6 (3.9%)	
Maximum burr size, mm			< 0.001*			< 0.001*
1.25	54 (17.7%)	60 (36.8%)		24 (15.7%)	53 (34.6%)	
1.5	161 (52.8%)	84 (51.5%)		78 (51.0%)	82 (53.6%)	
1.75	70 (23.0%)	17 (10.4%)		39 (25.5%)	16 (10.5%)	
2	20 (6.6%)	2 (1.2%)		12 (7.8%)	2 (1.3%)	
Large-diameter burr	90 (29.5%)	19 (11.7%)	< 0.001*	51 (33.3%)	18 (11.8%)	< 0.001*
Average stent diameter, mm	2.85 ± 0.59	2.77 ± 0.52	0.010*	2.93 ± 0.48	2.77 ± 0.54	< 0.001*
Total stent length, mm	70.08 ± 32.31	69.67 ± 31.86	0.762	68.16 ± 28.72	68.63 ± 31.58	0.887
Total number of stents	2.57 ± 1.20	2.55 ± 1.12	0.881	2.51 ± 1.08	2.51 ± 1.12	0.997
Contrast volume, ml	228.02 ± 66.19	204.60 ± 51.32	< 0.001*	228.46 ± 66.46	203.73 ± 50.72	0.002*
Operation time, min	101.09 ± 57.70	82.54 ± 43.99	< 0.001*	102.31 ± 71.04	81.06 ± 40.71	< 0.001*
Intraoperative use of adjunctive devices			0.779			0.226
ECMO	2 (0.7%)	1 (0.6%)		0 (0.0%)	1 (0.7%)	
IABP	32 (10.5%)	15 (9.2%)		18 (11.8%)	13 (8.5%)	
IABP+ECMO	3 (1.0%)	0 (0.0%)		2 (1.3%)	0 (0.0%)	
TPM	2 (0.7%)	0 (0.0%)		1 (0.7%)	0 (0.0%)	

Continuous variables are expressed as mean ± standard deviation (SD), and categorical variables are expressed as number (percentage) [n(%)]. Statistical comparisons were performed using the Wilcoxon rank-sum test for continuous variables and the χ² test (or Fisher’s exact test for variables with small expected frequencies) for categorical variables

*Abbreviations*: *IVI* Intravascular imaging, *CAG* Coronary angiography, *ECMO* Extracorporeal membrane oxygenation, *IABP* Intra-aortic balloon pump, *TPM* Temporary pacemaker, Large-diameter burr, Maximum rotational burr outer diameter ≥ 1.75 mm

*P < 0.05 indicates a statistically significant difference

After 1:1 PSM, 306 patients (153 per group) were included for the primary analysis. Baseline characteristics were well-balanced between the two groups, as confirmed by standardized mean differences all below 0.1 (*Supplementary Table S1*, *Supplementary Figure S1*), with only minor residual imbalance in a few variables. Procedural differences still persisted: large burr usage (33.3% vs. 11.8%), stent diameter (2.93 ± 0.48 mm vs. 2.77 ± 0.54 mm), contrast volume (228.46 ± 66.46 mL vs. 203.73 ± 50.72 mL), and operation time (102.31 ± 71.04 min vs. 81.06 ± 40.71 min; all *P* < 0.05; Tables 1 and 2). All covariates achieved acceptable balance in the stringent expanded PSM sensitivity cohort (*Supplementary Table S2*, *Supplementary Figure S2*).

### LASSO variable selection

To mitigate overfitting given the limited number of events, we performed variable selection using LASSO penalised Cox regression with 10-fold cross-validation in the PSM cohort. The treatment assignment (IVI- vs. CAG-guided) was prespecified for inclusion in the final model. From the remaining covariates, LASSO selected two predictors associated with MACE: the procedural period (2022–2024) and preoperative eGFR < 60 mL/(min·1.73 m²) (*Supplementary Figure S4*,* Table S3*).

### Clinical outcomes after PSM

**Fig. 2:**
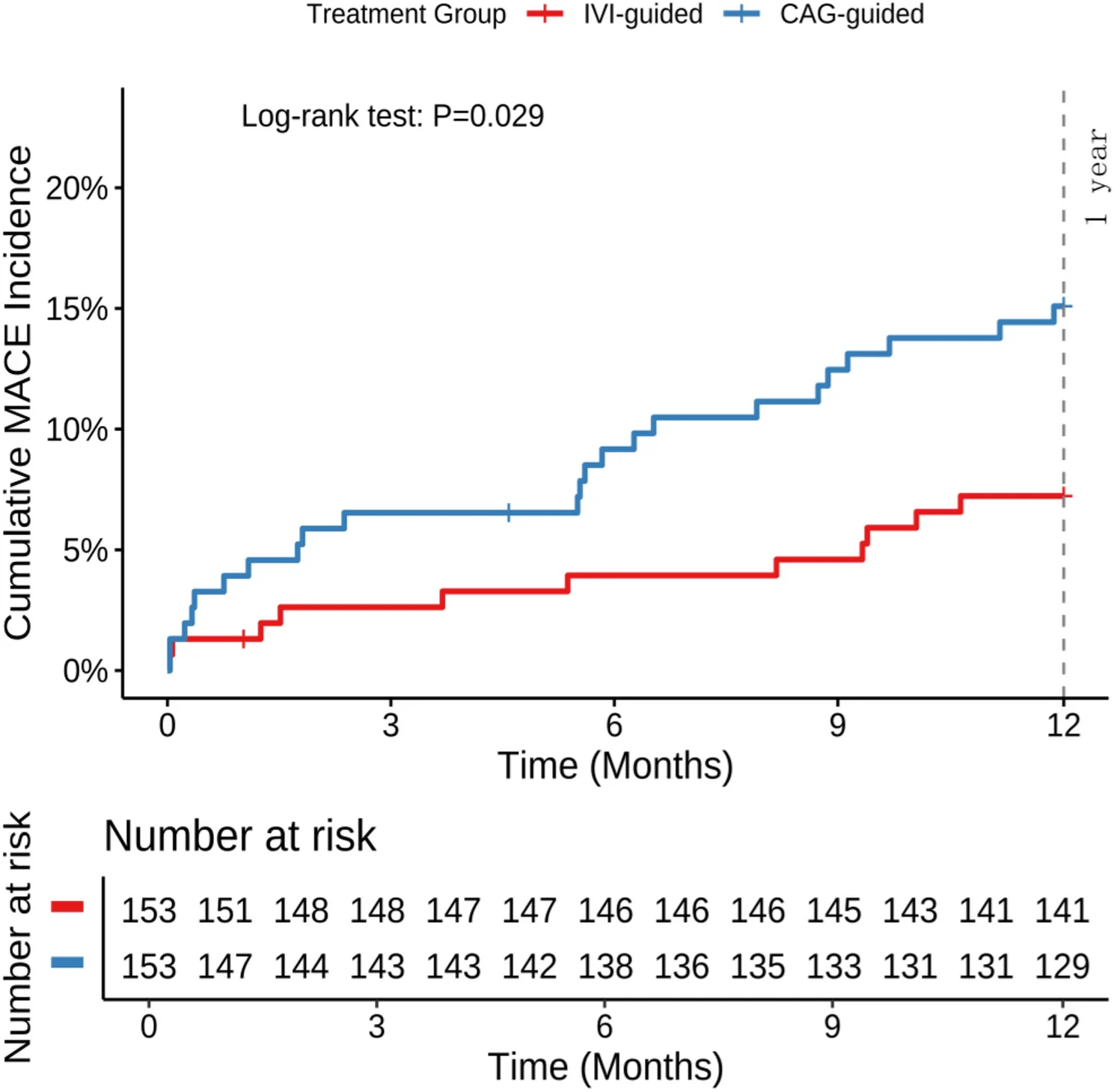
Kaplan-Meier survival curves comparing 12-month MACE incidence between IVI-guided and CAG-guided groups after PSM

Kaplan-Meier analysis demonstrated a significant separation in MACE-free survival between groups (Log-rank *P* = 0.029; Fig. 2). The cumulative incidence of MACE at 12 months was 15.0% in the CAG-guided group versus 7.2% in the IVI-guided group, corresponding to an unadjusted hazard ratio (HR) of 2.19 (95% CI: 1.07–4.48, *P* = 0.033) for the CAG-guided group.

For the primary adjusted analysis, we constructed a parsimonious multivariable Cox model to control for confounding while avoiding overfitting. A stratified Cox proportional hazards model (stratified by procedural period) was fitted. This adjusted model included the treatment group and the remaining LASSO-selected covariate, preoperative eGFR < 60 mL/(min·1.73 m²).

Results from the corresponding unstratified multivariable Cox model are presented in Table 3. Variance inflation factors for all covariates were below 5, indicating no substantial multicollinearity. Nevertheless, Schoenfeld residual testing identified a violation of the proportional hazards assumption for procedural period in this unstratified specification, justifying our stratified primary analysis.

**Table 3:** Multivariable Cox model for primary PSM cohort

Characteristic	*N*	Event *N*	HR	95% CI	*P*
Guidance group					
CAG	153	23	—	—	
IVI	153	11	0.42	0.21, 0.87	0.019
Procedural period					
2015–2018	108	6	—	—	
2019–2021	95	10	1.84	0.67, 5.12	0.239
2022–2024	103	18	3.51	1.39, 8.90	0.008
Preoperative eGFR < 60 mL/(min·1.73 m²)	57	12	2.46	1.21, 5.01	0.013

This unstratified Cox model incorporates procedural period as an adjusted covariate, Schoenfeld residual testing confirming non-proportional hazards for this variable. The primary prespecified analytical model shown in Table 4 adopts a stratified Cox framework grouped by procedural period to resolve this assumption violation. Data are reported as hazard ratio (HR) with 95% confidence interval (CI). Two-sided *P* < 0.05 denotes statistical significance

In this primary stratified adjusted model, IVI guidance remained independently associated with a significantly lower risk of 12-month MACE (adjusted HR = 0.42, 95% CI: 0.20–0.86, *P* = 0.018). Preoperative eGFR < 60 mL/(min·1.73 m²) was also an independent predictor of MACE (Table 4).

**Table 4:** Stratified multivariate Cox regression model stratified by procedural period (primary analytical model)

Characteristic	*N*	Event *N*	HR	95% CI	*P*
Guidance group					
CAG	153	23	—	—	—
IVI	153	11	0.42	0.20, 0.86	0.018
Preoperative eGFR					
≥ 60 mL/(min·1.73 m²)	249	22	—	—	—
< 60 mL/(min·1.73 m²)	57	12	2.52	1.24, 5.13	0.011

This primary multivariable Cox model was stratified by 3-category procedural period to address non-proportional hazards identified via Schoenfeld residual testing. Adjusted covariates were two LASSO-screened predictors: IVI guidance group and preoperative eGFR < 60 mL/(min·1.73 m²). Results are presented as hazard ratio (HR) with 95% confidence interval (CI). Two-sided *P* < 0.05 was defined as statistically significant

*Abbreviations*: *LASSO* Least absolute shrinkage and selection operator

The association between IVI guidance and lower 12-month MACE risk remained consistent across all predefined sensitivity analyses. When the identical stratified Cox proportional hazards framework was applied to the more stringently balanced expanded PSM sensitivity cohort (147 paired patients per group), IVI guidance was similarly associated with reduced MACE risk (HR = 0.37, 95% CI: 0.16–0.82, *P* = 0.014). The consistent direction, comparable effect magnitude, and sustained statistical significance of hazard ratios across two distinct matched cohorts reinforce the robustness of our primary observational findings. Full outputs for all sensitivity analyses are provided in *Supplementary Tables S4–S5* and *Supplementary Figures S2–S3*,* S7*.

Regarding secondary endpoints, the composite acute myocardial infarction (AMI) endpoint occurred in 4 patients: 1 (0.7%) in the IVI-guided group and 3 (2.0%) in the CAG-guided group. Given the limited number of events, no between-group statistical comparison was performed. Incidence rates of cardiac death and clinically driven target-lesion revascularization are presented in Table 5.

**Table 5:** 12-month clinical outcomes after PSM [n (%)]

Outcome	IVI (*n* = 153)	CAG (*n* = 153)	HR (95%CI)	*P*
MACE	11(7.2%)	23(15%)	2.19 (1.07–4.48)	0.033*
Cardiac death	3(2%)	13(8.5%)	4.45 (1.27–15.61)	0.020*
Clinically Driven TLR	8(5.2%)	6(3.9%)	0.79 (0.27–2.28)	0.666
All-cause death	4(2.6%)	18(11.8%)	4.66 (1.58–13.77)	< 0.005*
Clinically Driven TVR	7(4.6%)	5(3.3%)	0.76 (0.24–2.39)	0.635
AMI**	1(0.7%)	3(2%)	3.14 (0.33–30.23)	0.321

*Abbreviations*: *HR* Hazard ratio, *CI* Confidence interval, *MACE* Major adverse cardiac events, *TVR* Target vessel revascularization, *TLR* Target lesion revascularization, *AMI* acute myocardial infarction

*P < 0.05 indicates statistical significance

**Due to small event numbers, target vessel myocardial infarction, perioperative myocardial infarction, spontaneous myocardial infarction, and non-target vessel myocardial infarction were combined into acute myocardial infarction (AMI)

## Discussion

In this study, IVI-guided RA was associated with lower 12-month MACE and mortality than CAG-guided RA. This association remained consistent across sensitivity analyses, and comparable procedural success mitigated concerns about selection bias. Collectively, these observational data support the potential clinical utility of IVI guidance in the RA setting.

Our results extend the existing evidence base for IVI in complex PCI. Although previous meta-analyses have established the superiority of IVI over angiography in reducing adverse events across broad PCI populations [13], large-scale data specifically focused on the RA subset have been scarce. This study addresses that critical gap by demonstrating that the prognostic associations of IVI are not only preserved but are particularly pronounced in the context of RA—a scenario characterised by challenging calcific plaque morphology [3]. Mechanistically, IVI facilitates superior procedural planning and execution in calcified lesions. It enables accurate assessment of vessel size, calcium distribution (arc, thickness, and length), and lesion length, thereby aiding optimal burr size selection and strategy for plaque modification [2, 3]. This likely translates into more effective calcium ablation, fewer procedural complications such as dissection or perforation, and ultimately, a more favorable milieu for stent implantation [3].

The temporal pattern of outcome separation is noteworthy. The Kaplan–Meier curves for MACE began to diverge markedly after the peri-procedural period, with the maximum difference apparent around six months. This pattern is consistent with a hypothesis that the observed association between IVI guidance and lower long-term adverse event risk may relate more to the mitigation of later-term events (e.g., stent thrombosis, death from progressive heart failure) rather than the prevention of acute procedural complications. One plausible mechanistic explanation for this pattern involves the optimisation of stent implantation. Suboptimal stent expansion and lesion coverage are established risk factors for late adverse events, and this risk is particularly heightened in calcified lesions when guided by angiography alone. Studies such as CLI-OPCI II have shown that IVI-derived metrics of suboptimal stenting strongly predict device-oriented cardiovascular events on long-term follow-up [14]. Thus, it is conceivable that IVI guidance during RA, by facilitating more adequate stent expansion and lesion coverage, may attenuate these modifiable late risks. Furthermore, it is hypothesized that achieving more complete and effective revascularization may confer systemic benefits, such as ameliorating residual ischemia and microvascular dysfunction, potentially leading to reverse ventricular remodeling and a lower incidence of fatal arrhythmias over time—a hypothesis supported by long-term follow-up data from trials like IVUS-XPL [15]. However, these mechanistic explanations remain speculative, and the present retrospective study design cannot establish or validate such causal pathways.

An intriguing finding that warrants explanation is the lack of a significant difference in target lesion revascularization (TLR) rates between groups, despite clear mortality associations with IVI. We propose that this may reflect a “competing risk” phenomenon [6]. The substantially higher mortality rate in the CAG-guided group (11.8% vs. 2.6%) implies that a number of patients who might have developed clinically significant restenosis did not survive long enough to require or undergo repeat revascularisation. Conversely, the improved survival in the IVI group allowed for the potential detection and treatment of non-fatal restenosis events [4, 16]. This interpretation is consistent with the high-risk profile of our study population and underscores that mortality, rather than repeat revascularisation, is the most critical endpoint in this setting.

Our study identified a temporal trend of increasing MACE incidence in later procedural years. Because the proportional hazards assumption was violated for the procedural period variable, we employed a stratified Cox regression model to account for this time-dependent confounding in our primary analysis. This trend likely reflects the evolution of clinical practice toward treating increasingly complex patient profiles and coronary lesions over time. Supporting this interpretation, the prevalence of high-risk coronary anatomy (e.g., multivessel disease, chronic total occlusions, severe calcification) increased modestly across the study periods (from 91.5% to 92.8%), paralleled by a steady rise in the clinical adoption of intravascular imaging (IVI) guidance (from 56.9% to 70.5%).Crucially, after stratification by procedural period and adjustment for other covariates, IVI guidance remained independently associated with a significantly reduced risk of MACE (adjusted HR = 0.42, 95% CI: 0.20 to 0.86, *P* = 0.018). This robust finding suggests that the procedural benefit associated with IVI is substantial and may effectively mitigate the elevated baseline risk inherent in contemporary, more complex interventional practice.

Our analysis also reaffirmed the prognostic importance of baseline renal function, identifying preoperative eGFR < 60 mL/(min·1.73 m²) as an independent predictor of MACE, consistent with prior registry data [1]. Importantly, after matching, the prevalence of renal impairment was balanced between groups, yet an inverse association between IVI guidance and 12-month adverse events remained evident despite higher contrast volumes in the IVI group. A potential explanation for this observation is that more thorough lesion preparation achievable with IVI guidance might counterbalance the theoretical risks associated with higher contrast volume. However, this causal mechanism remains hypothetical and cannot be confirmed by our observational data.

We acknowledge several limitations inherent to our study design. First, residual and unmeasured confounding cannot be fully eliminated despite rigorous statistical adjustments including PSM, LASSO variable selection, and stratified Cox regression; unrecorded confounders such as inter-operator variation and subtle plaque morphological features may still influence our observational associations. Second, the sample size after matching limited statistical power for subgroup analyses of individual myocardial infarction subtypes, given the low event count of discrete MI endpoints. Third, to ensure that our comparison reflected the efficacy of imaging guidance rather than procedural feasibility, we excluded patients with rotational atherectomy failure prior to matching. However, the nearly identical procedural success rates between groups (93.1% vs. 92.2%; *P* = 0.820) suggest that this exclusion is unlikely to have introduced substantial selection bias. Fourth, this study combined IVUS and OCT data into a unified IVI cohort without head-to-head subgroup comparison, and no standardised intraprocedural imaging protocols were implemented across participating centres. Uniform criteria for calcium quantification, burr sizing, and adequate stent expansion were absent, with all imaging decisions left to operator discretion. OCT and IVUS possess distinct technical strengths: OCT provides superior resolution for calcium characterisation [17], whereas IVUS offers better penetration in dense calcium [18], yet the inter-modality comparison was not feasible under this heterogeneous procedural framework. Future prospective registries with unified imaging standards or randomized trials are required to validate our results and clarify differential associations between the two imaging modalities. Finally, all participants were recruited from Chinese cardiac centres, which may limit the generalisability of our findings to other ethnic groups or international clinical populations.

Interpretation of the Effect Size: It is important to contextualise our primary finding (adjusted HR = 0.42). Our study exclusively enrolled a high-risk population with severely calcified lesions requiring rotational atherectomy, in whom the baseline risk of MACE is substantially higher than in general PCI populations. In such complex cases, the absolute prognostic benefit associated with an effective adjunctive tool like intravascular imaging is likely magnified, which may explain the greater magnitude of risk reduction observed here compared with broader PCI registry data. Nonetheless, the limitations outlined above underscore that our results, while robust within the constraints of this retrospective design, should be validated in prospective, randomized trials.

In conclusion, this multicentre study provides observational evidence that IVI guidance during rotational atherectomy for severely calcified coronary lesions is associated with significantly better 12-month clinical outcomes, including a substantial reduction in mortality. These findings highlight the potential importance of intravascular imaging in this setting and call for further validation in prospective, randomized trials. 

## Supplementary Information

Supplementary Material 1.

## Data Availability

Data availability statementAll clinical datasets analysed in this retrospective multicentre observational study cannot be publicly deposited or released online, due to local hospital patient privacy laws and institutional data governance policies that prohibit unrestricted sharing of identifiable individual clinical records. Only fully de-identified, anonymized aggregate patient data can be provided. Qualified researchers may submit a formal data access request to the corresponding author; data sharing requires written institutional approval from both the applicant’s institution and our participating medical centres. No sequencing, omics or publicly archived omics datasets (e.g., GEO, SRA) were generated or analysed for this work.

## Abbreviations

AMI: Acute myocardial infarction
ACC/AHA: American College of Cardiology/American Heart Association
ACS: Acute coronary syndrome
CAG: Coronary angiography
CABG: Coronary artery bypass grafting
CTO: Chronic total occlusion
DAPT: Dual antiplatelet therapy
DES: Drug-eluting stent
ECMO: Extracorporeal membrane oxygenation
eGFR: Estimated glomerular filtration rate
HR: Hazard ratio
CI: Confidence interval
IABP: Intra-aortic balloon pump
IVI: Intravascular imaging
IVUS: Intravascular ultrasound
LASSO: Least Absolute Shrinkage and Selection Operator
LVEF: Left ventricular ejection fraction
MACE: Major adverse cardiac events
OCT: Optical coherence tomography
PCI: Percutaneous coronary intervention
PSM: Propensity score matching
RA: Rotational atherectomy
SD: Standard deviation
TIMI: Thrombolysis in Myocardial Infarction
TLR: Target lesion revascularization
TPM: Temporary pacemaker
TV-MI: Target vessel myocardial infarction
TVR: Target vessel revascularization
