# Supplementary material for: Comparison between angiography or intravascular imaging guided coronary rotational atherectomy
Source: BMC Cardiovasc Disord. 2026 Aug 11;26:1075. doi: 10.1186/s12872-026-06411-6 (PMC13757062; doi:10.1186/s12872-026-06411-6)
Supplement: Supplementary file 1 [file 12872_2026_6411_MOESM1_ESM.docx]

**Supplement material**

**Comparison between angiography or intravascular imaging guided coronary rotational atherectomy**

**Sensitivity Analyses**

1. Expanded stringent PSM cohort replication

To test the reproducibility of our primary endpoint results, we replicated the unstratified 3-covariate Cox regression model in a stringent expanded propensity score-matched cohort, comprising 147 matched CAG/IVI pairs (294 total patients, 28 adjudicated MACE events). Consistent with the main analysis, the model was adjusted for IVI guidance, procedural calendar period, and preoperative eGFR < 60 mL/(min·1.73 m²). Complete regression outputs for this sensitivity cohort are shown in *Supplementary Table S4*.

*Supplementary Table S5* directly compared HR and 95% CI values derived from the primary and expanded PSM Cox models. The inverse association of IVI guidance remained consistent in magnitude and statistical significance across both matched cohorts (HR = 0.37, 95% CI: 0.16–0.82, P = 0.014), verifying the robustness of our primary findings.

2. Temporal confounding and collinearity diagnostics

Temporal confounding and multicollinearity diagnostics Cumulative hazard curves stratified by three procedural periods (*Supplementary Figure S7*) demonstrated progressively elevated long-term MACE risk across successive calendar eras, confirming substantial temporal imbalance between the two groups and formal violation of the proportional hazards assumption for the variable of procedural period. Multicollinearity assessment via variance inflation factor (VIF) plots was presented in *Supplementary Figure S4B*; all candidate predictors yielded VIF values below 5, ruling out severe multicollinearity that would distort effect estimates. *Supplementary Table S7* further verified the robustness of the protective hazard ratio associated with IVI guidance across alternative time-stratified regression frameworks in sensitivity analyses.

3. Procedural success subgroup analysis

Overall RA procedural success rates were comparable between CAG-guided and IVI-guided groups (93.14% vs. 92.15%, P = 0.820, *Supplementary Figure S6*). Subgroup breakdowns of success rates for IVUS-only, OCT-only, and dual IVUS+OCT guidance were summarized in *Supplementary Table S6*. The balanced procedural success rates ruled out selection bias arising from exclusion of patients with RA procedural failure.

4. Survival and covariate balance diagnostics for sensitivity cohort

Kaplan-Meier MACE curves for the expanded stringent PSM cohort were provided in *Supplementary Figure S3*, showing consistent intergroup event separation consistent with the primary cohort survival analysis. The Love plot for the sensitivity PSM cohort (*Supplementary Figure S2*) demonstrated all covariates achieved absolute SMD < 0.1 after matching, with complete SMD values listed in *Supplementary Table S2*. Temporal patient distribution across procedural periods was visualized in *Supplementary Figure S5*.

**Supplementary Abbreviations**

CAG, coronary angiography; CI, confidence interval; eGFR, estimated glomerular filtration rate; HR, hazard ratio; IVI, intravascular imaging; IVUS, intravascular ultrasound; LASSO, least absolute shrinkage and selection operator; MACE, major adverse cardiac events; OCT, optical coherence tomography; PSM, propensity score matching; RA, rotational atherectomy; SMD, standardized mean difference; VIF, variance inflation factor.

**Supplementary Table**

**Supplementary Table S1 Baseline Characteristics Balance before and after primary Propensity Score Matching**

| **Characteristics** | **Before PSM** | | | | **PSM** | | | |
| --- | --- | --- | --- | --- | --- | --- | --- | --- |
|  | **IVI**  **n = 305** | **CAG**  **n = 163** | **P** | **SMD** | **IVI**  **n = 153** | **CAG**  **n = 153** | **P** | **SMD** |
| Age, y | 72 ± 8 | 73 ± 9 | 0.139 | 0.124 | 72 ± 8 | 73 ± 8 | 0.373 | 0.121 |
| Male | 193 (63%) | 98 (60%) | 0.502 | 0.064 | 100 (65%) | 93 (61%) | 0.407 | 0.094 |
| Hypertension | 238 (78%) | 124 (76%) | 0.63 | 0.046 | 119 (78%) | 117 (77%) | 0.785 | 0.031 |
| Diabetes mellitus | 117 (38%) | 63 (39%) | 0.951 | 0.006 | 49 (32%) | 59 (39%) | 0.232 | 0.134 |
| Dyslipidemia | 177 (58%) | 105 (64%) | 0.252 | 0.113 | 80 (52%) | 96 (63%) | 0.064 | 0.216 |
| Previous Renal Insufficiency | 59 (19%) | 38 (23%) | 0.313 | 0.094 | 26 (17%) | 31 (20%) | 0.463 | 0.081 |
| Previous dialysis | 7 (2%) | 5 (3%) | 0.76 | 0.045 | 2 (1%) | 5 (3%) | 0.448 | 0.11 |
| Previous myocardial infarction | 29 (10%) | 18 (11%) | 0.599 | 0.049 | 12 (8%) | 15 (10%) | 0.545 | 0.066 |
| Previous PCI | 79 (26%) | 44 (27%) | 0.798 | 0.025 | 39 (26%) | 42 (28%) | 0.697 | 0.044 |
| Previous CABG | 4 (1%) | 1 (1%) | 0.662 | 0.089 | 1 (1%) | 0 (0%) | >0.999 | 0.115 |
| Previous peripheral vascular disease | 19 (6%) | 16 (10%) | 0.16 | 0.121 | 12 (8%) | 16 (11%) | 0.428 | 0.085 |
| Current smoking | 65 (21%) | 38 (23%) | 0.619 | 0.047 | 37 (24%) | 35 (23%) | 0.788 | 0.031 |
| Clinical syndrome |  |  | 0.731 | 0.033 |  |  | 0.515 | 0.072 |
| ACS | 260 (85%) | 137 (84%) |  |  | 133 (87%) | 129 (84%) |  |  |
| Stable angina pectoris | 45 (15%) | 26 (16%) |  |  | 20 (13%) | 24 (16%) |  |  |
| Preoperative LVEF, % | 59 ± 10 | 58 ± 10 | 0.220 | 0.066 | 59 ± 10 | 59 ± 10 | 0.609 | 0.007 |
| Preoperative eGFR, ml/(min·1.73 m²) | 77 ± 23 | 75 ± 24 | 0.140 | 0.107 | 78 ± 21 | 76 ± 23 | 0.467 | 0.089 |
| Procedure period  (3 categories) |  |  | 0.028 |  |  |  | 0.149 |  |
| 2015–2018 | 87(29%) | 66 (41%) |  | 0.244 | 46(30%) | 62 (41%) |  | 0.213 |
| 2019–2021 | 101(33.%) | 48(29.4%) |  | 0.080 | 50(32.7%) | 45(29%) |  | 0.072 |
| 2022–2024 | 117.0(38%) | 49.0 (30%) |  | 0.181 | 57(37%) | 46(30%) |  | 0.157 |
| *Angiographic Baseline Data* |  |  |  |  |  |  |  |  |
| Multivessel lesions | 276 (91%) | 151 (93%) | 0.434 | 0.082 | 137 (90%) | 141 (92%) | 0.428 | 0.097 |
| Bifurcation lesions | 129 (42%) | 51 (31%) | 0.020* | 0.237 | 46 (30%) | 45 (29%) | 0.900 | 0.014 |
| CTO lesions | 38 (12%) | 38 (23%) | 0.002* | 0.257 | 29 (19%) | 28 (18%) | 0.883 | 0.017 |
| Target vessel |  |  |  |  |  |  |  |  |
| LM | 17 (6%) | 9 (6%) | 0.981 | 0.002 | 7 (5%) | 8 (5%) | 0.791 | 0.029 |
| LAD | 236 (77%) | 121 (74%) | 0.446 |  | 114 (75%) | 115 (75%) | 0.895 |  |
| LCX | 19 (6%) | 8 (5%) | 0.559 |  | 9 (6%) | 8 (5%) | 0.803 |  |
| RCA | 55(18%) | 34(21%) | 0.458 |  | 31(20%) | 30(20%) | 0.886 |  |

Notes: Data are presented as mean ± standard deviation for continuous variables and number (percentage) for categorical variables. The standardized mean difference (SMD) was used to quantify covariate balance before and after propensity score matching (PSM), with an absolute SMD value < 0.1 considered indicative of adequate balance. Group comparisons were performed using the Wilcoxon rank-sum test for continuous variables and the χ² test or Fisher’s exact test for categorical variables, as appropriate.

Abbreviations: CAG, angiography-only guided rotational atherectomy; IVI, intravascular imaging-guided rotational atherectomy (combining IVUS and OCT); PSM, propensity score matching; SMD, standardized mean difference.

**Supplementary Table S2 Baseline Characteristics** **balance before and after stringent expanded PSM for sensitivity analysis**

| **Characteristics** | **Before PSM** | | | | **PSM** | | | |
| --- | --- | --- | --- | --- | --- | --- | --- | --- |
|  | **IVI**  **n = 305** | **CAG**  **n = 163** | **P** | **SMD** | **IVI**  **n = 147** | **CAG**  **n = 147** | **P** | **SMD** |
| Age, y | 72 ± 8 | 73 ± 9 | 0.139 | 0.124 | 73 ± 7 | 73 ± 9 | 0.373 | 0.006 |
| Male | 193(63%) | 98 (60%) | 0.502 | 0.064 | 97 (66%) | 90 (61%) | 0.407 | 0.097 |
| Hypertension | 238(78%) | 124(76%) | 0.63 | 0.046 | 111 (76%) | 111 (76%) | 0.785 | 0.000 |
| Diabetes mellitus | 117(38%) | 63 (39%) | 0.951 | 0.006 | 52 (35%) | 58 (39%) | 0.232 | 0.084 |
| Dyslipidemia | 177(58%) | 105(64%) | 0.252 | 0.113 | 84 (57%) | 89 (61%) | 0.064 | 0.071 |
| Previous Renal Insufficiency | 59 (19%) | 38 (23%) | 0.313 | 0.094 | 26 (17%) | 31 (20%) | 0.463 | 0.081 |
| Previous dialysis | 7 (2%) | 5 (3%) | 0.76 | 0.045 | 2 (1%) | 5 (3%) | 0.448 | 0.110 |
| Previous myocardial infarction | 29 (10%) | 18 (11%) | 0.599 | 0.049 | 19 (13%) | 15 (10%) | 0.545 | 0.087 |
| Previous PCI | 79 (26%) | 44 (27%) | 0.798 | 0.025 | 41 (28%) | 39 (27%) | 0.697 | 0.031 |
| Previous CABG | 4 (1%) | 1 (1%) | 0.662 | 0.089 | 1 (1%) | 1 (1%) | >0.999 | 0.000 |
| Previous peripheral vascular disease | 19 (6%) | 16 (10%) | 0.160 | 0.121 | 11 (8%) | 12 (8%) | 0.428 | 0.023 |
| Current smoking | 65 (21%) | 38 (23%) | 0.619 | 0.047 | 37 (25%) | 32 (22%) | 0.788 | 0.080 |
| Clinical syndrome |  |  | 0.731 | 0.033 |  |  | 0.515 | 0.056 |
| ACS | 260(85%) | 137(84%) |  |  | 124(84%) | 121(82%) |  |  |
| Stable angina pectoris | 45 (15%) | 26 (16%) |  |  | 23 (16%) | 26 (18%) |  |  |
| Preoperative LVEF, % | 59 ± 10 | 58 ± 10 | 0.220 | 0.066 | 59 ± 10 | 59 ± 10 | 0.609 | 0.000 |
| Preoperative eGFR, ml/(min·1.73 m²) | 77 ± 23 | 75 ± 24 | 0.140 | 0.107 | 78 ± 22 | 77 ± 22 | 0.467 | 0.024 |
| Procedure period  (3 categories) |  |  | 0.028 |  |  |  | 0.149 |  |
| 2015–2018 | 87(29%) | 66 (41%) |  | 0.244 | 46 (30%) | 62 (41%) |  | 0.305 |
| 2019–2021 | 101(33%) | 48(29%) |  | 0.080 | 50 (33%) | 45 (29%) |  | 0.000 |
| 2022–2024 | 117(38%) | 49(30%) |  | 0.181 | 57 (37%) | 46 (30%) |  | 0.327 |
| *Angiographic Baseline Data* |  |  |  |  |  |  |  |  |
| Multivessel lesions | 276(91%) | 151(93%) | 0.434 | 0.082 | 132(90%) | 135 (92%) | 0.428 | 0.078 |
| Bifurcation lesions | 129(42%) | 51 (31%) | 0.020* | 0.237 | 47 (32%) | 47 (32%) | 0.900 | 0.000 |
| CTO lesions | 38 (12%) | 38 (23%) | 0.002* | 0.257 | 28 (19%) | 26 (18%) | 0.883 | 0.032 |
| Target vessel |  |  |  |  |  |  |  |  |
| LM | 17 (6%) | 9 (6%) | 0.981 | 0.002 | 8 (5%) | 6 (4%) | 0.791 | 0.060 |
| LAD | 236(77%) | 121(74%) | 0.446 |  | 114(75%) | 115 (75%) | 0.895 |  |
| LCX | 19 (6%) | 8 (5%) | 0.559 |  | 9 (6%) | 8 (5%) | 0.803 |  |
| RCA | 55(18%) | 34(21%) | 0.458 |  | 31 (20%) | 30 (20%) | 0.886 |  |

**Supplementary Table S3 Collinearity assessment, univariate Cox analyses, LASSO variable selection and multivariable Cox regression in the primary propensity score-matched cohort**

Part 1 Univariate Cox analysis

| **Variable** | **Univariate Analysis** | | |
| --- | --- | --- | --- |
|  | **HR** | **95% CI** | **P** |
| Angiography Guidance | 2.19 | 1.07-4.48 | 0.033* |
| Age > 75 years | 1.72 | 0.88-3.38 | 0.113 |
| Gender (Male) | 0.81 | 0.41-1.59 | 0.535 |
| Current smoking | 0.99 | 0.45-2.19 | 0.983 |
| Hypertension | 0.82 | 0.38-1.76 | 0.612 |
| Diabetes mellitus | 1.15 | 0.58-2.30 | 0.689 |
| Dyslipidemia | 1.58 | 0.77-3.23 | 0.215 |
| Previous peripheral vascular disease | 1.77 | 0.68-4.56 | 0.240 |
| Previous PCI | 0.99 | 0.46-2.12 | 0.979 |
| Previous CABG | 0.00 | 0.00-Inf | 0.997 |
| Previous myocardial infarction | 2.43 | 1.01-5.87 | 0.048* |
| Clinical syndrome (ACS) | 2.82 | 0.68-11.77 | 0.155 |
| Preoperative eGFR < 60 ml/(min·1.73 m²) | 2.62 | 1.29-5.29 | 0.007* |
| Preoperative LVEF<50% | 1.56 | 0.71-3.44 | 0.273 |
| Large-diameter burr | 1.04 | 0.47-2.29 | 0.926 |
| CTO lesions | 1.93 | 0.92-4.03 | 0.081 |
| Bifurcation lesions | 1.12 | 0.55-2.30 | 0.751 |
| Multivessel lesions | 1.66 | 0.40-6.95 | 0.484 |
| LM | 2.15 | 0.66-7.02 | 0.207 |
| LAD | 0.60 | 0.30-1.21 | 0.155 |
| LCX | 1.75 | 0.53-5.72 | 0.355 |
| RCA | 1.46 | 0.68-3.13 | 0.330 |
| Total number of stents | 1.12 | 0.84-1.49 | 0.444 |
| Total stent length, mm | 1.00 | 0.99-1.01 | 0.651 |
| Average stent diameter, mm | 1.12 | 0.55-2.30 | 0.751 |
| Contrast volume, ml | 1.00 | 0.99-1.00 | 0.784 |
| Operation time, min | 1.00 | 1.00-1.01 | 0.455 |
| Vascular access (Dual arterial access) | 1.72 | 0.53-5.62 | 0.370 |
| Procedure period |  |  |  |
| 2015–2018 | — | — |  |
| 2019–2021 | 1.92 | 0.70-5.27 | 0.208 |
| 2022–2024 | 3.25 | 1.29-8.19 | 0.012* |

Notes: Unadjusted Cox regression was performed for all candidate clinical and procedural covariates to screen potential prognostic predictors. HR and 95% CI are presented for each variable. *P < 0.05 indicates statistical significance.Large-diameter burr,Maximum rotational burr outer diameter ≥1.75 mm.

Abbreviations: CI, confidence interval; HR, hazard ratio.

Part 2 Collinearity diagnosis

| **Term** | **VIF** | **Tolerance** |
| --- | --- | --- |
| Large diameter burr | 1.127424 | 0.8869778 |
| Contrast volume (mL) | 1.148666 | 0.8705753 |
| Operation time (min) | 1.097955 | 0.9107842 |
| Average stent diameter (mm) | 1.065639 | 0.9384045 |
| Procedure period | 1.166337 | 0.8573853 |
| Dyslipidemia | 1.114788 | 0.8970317 |
| Vascular access_Single arterial | 1.055900 | 0.9470594 |
| Diabetes mellitus | 1.046969 | 0.9551381 |
| Previous CABG | 1.000000 | 1.0000000 |
| Age | 1.094607 | 0.9135702 |
| Preoperative eGFR＜60 ml/(min·1.73 m²) | 1.134620 | 0.8813525 |
| Previous myocardial infarction | 1.112017 | 0.8992671 |

Notes: VIF (Variance Inflation Factor) < 5 and Tolerance > 0.1 for all variables indicated no severe multicollinearity among the candidate covariate pool. Inf, infinite value.Abbreviations: SE, standard error; VIF, variance inflation factor.

Part 3 LASSO regression non-zero coefficients

| **Variable** | **Coefficient** |
| --- | --- |
| Large diameter burr ≥1.75 mm | 0 |
| Contrast volume (mL) | 0 |
| Operation time (min) | 0 |
| Average stent diameter (mm) | 0 |
| Procedure period 2019–2021 | 0 |
| Procedure period 2022–2024 | 0.4400619 |
| Dyslipidemia | 0 |
| Single arterial | 0 |
| Diabetes mellitus | 0 |
| Previous CABG | 0 |
| Age | 0 |
| Preoperative eGFR＜60 ml/(min·1.73 m²) | 0.5845905 |
| Previous myocardial infarction | 0.3095329 |

LASSO non-zero coefficients selected via 10-fold cross validation.Candidate variables for LASSO: post-PSM covariates with univariate P<0.05 or residual SMD>0.1.

Part 4 Unstratified multivariable Cox (Primary Model)

| **Characteristic** | **N** | **Event N** | **HR** | **95% CI** | **P** |
| --- | --- | --- | --- | --- | --- |
| Guidance.group |  |  |  |  |  |
| CAG | 153 | 23 | — | — |  |
| IVI | 153 | 11 | 0.42 | 0.21, 0.87 | 0.019 |
| Procedure.period |  |  |  |  |  |
| 2015–2018 | 108 | 6 | — | — |  |
| 2019–2021 | 95 | 10 | 1.84 | 0.67, 5.12 | 0.239 |
| 2022–2024 | 103 | 18 | 3.51 | 1.39, 8.90 | 0.008 |
| Preoperative eGFR < 60 ml/(min·1.73 m²) | 57 | 12 | 2.46 | 1.21, 5.01 | 0.013 |

Notes: A parsimonious Cox model was constructed to respect the ≥10 events-per-variable (EPV) guideline (34 MACE events). The treatment group (IVI vs. CAG) was forced into the model. Procedural period and preoperative eGFR < 60 ml/(min·1.73 m²) were selected via LASSO regression (10-fold cross-validation) from other covariates for adjustment.

**Supplementary Table S4 Multivariable Cox regression analysis of expanded PSM cohort for sensitivity analysis**

| **Characteristic** | **N** | **Event N** | **HR** | **95% CI** | **P** |
| --- | --- | --- | --- | --- | --- |
| Guidance group |  |  |  |  |  |
| CAG | 147 | 19 | — | — |  |
| IVI | 147 | 9 | 0.37 | 0.16, 0.82 | 0.014 |
| Procedure period |  |  |  |  |  |
| 2015–2018 | 96 | 4 | — | — |  |
| 2019–2021 | 88 | 6 | 1.74 | 0.49, 6.17 | 0.394 |
| 2022–2024 | 110 | 18 | 5.15 | 1.73, 15.35 | 0.003 |
| Preoperative eGFR < 60 ml/(min·1.73 m²) | 58 | 8 | 1.83 | 0.80, 4.19 | 0.150 |

Notes: This table presents outputs from an unstratified Cox model fitted within the expanded full-covariate PSM sensitivity cohort (n = 294, 147 matched pairs per group; total 28 MACE events). The identical three covariates (IVI guidance group, procedural period, preoperative eGFR < 60 mL/[min·1.73 m²]) were adjusted as confounding factors, consistent with the unstratified model for the primary cohort shown in *Table 4*. Schoenfeld residual testing confirmed non-proportional hazards for procedural period, so stratified Cox regression (*Table 5* in main text) served as the primary analytical framework rather than this unstratified specification. Full cross-cohort comparisons of hazard ratios from this sensitivity model and the primary unstratified model are summarized in *Supplementary Table S5.*

Abbreviations:EPV, events per variable; LASSO, least absolute shrinkage and selection operator; MACE, major adverse cardiac events.

**Supplementary Table S5 Comparison of primary and sensitivity multivariable Cox regression models after PSM**

| **Variable** | **Primary Model**  **HR (95%CI)** | **P** | **Sensitivity Model**  **HR (95%CI)** | **P** |
| --- | --- | --- | --- | --- |
| Guidance.group |  |  |  |  |
| CAG | — |  | — |  |
| IVI | 0.42 (0.21–0.87) | 0.019 | 0.37 (0.16–0.82) | 0.014 |
| Procedure.period |  |  |  |  |
| 2015–2018 | — |  | — |  |
| 2019–2021 | 1.84 (0.67–5.12) | 0.239 | 1.74 (0.49–6.17) | 0.394 |
| 2022–2024 | 3.51 (1.39–8.90) | 0.008 | 5.15 (1.73–15.35) | 0.003 |
| Preoperative eGFR < 60 ml/(min·1.73 m²) | 2.46 (1.21–5.01) | 0.013 | 1.83 (0.80–4.19) | 0.150 |

**Supplementary Table S6 Procedural success rates stratified by guidance modality and intravascular imaging subtype**

Part A: Overall procedural success by guidance strategy

| **Guidance strategy** | **Total procedures, n** | **Successful procedures, n** | **Failure procedures, n** | **Success rate, %** | **Failure rate, %** |
| --- | --- | --- | --- | --- | --- |
| CAG | 175 | 163 | 12 | 93.14 | 6.86 |
| IVI | 331 | 305 | 26 | 92.15 | 7.85 |
| Overall | 506 | 468 | 38 | 92.49 | 7.51 |

Part B: Procedural success stratified by intravascular imaging subtype

| **IVI subgroup** | **Total procedures,n** | **Successful procedures,n** | **Success rate,%** |
| --- | --- | --- | --- |
| Dual IVUS+OCT | 10 | 10 | 100.00 |
| IVUS-only | 239 | 219 | 91.63 |
| OCT-only | 82 | 76 | 92.68 |

Part C: Chi-square test for inter-subgroup comparison of procedural success

| **Comparison** | **χ² statistic** | **Degrees of freedom** | **P** |
| --- | --- | --- | --- |
| IVI subgroup procedural success (IVUS-only vs OCT-only vs dual IVUS+OCT ) | 0.972 | 2 | 0.615 |

Notes: Chi-square tests were used to compare procedural success across groups. The overall cohort included 506 patients undergoing rotational atherectomy (RA). Subgroup analyses within the IVI cohort compared success rates among patients receiving IVUS-only, OCT-only, and combined dual IVUS+OCT guidance. No statistically significant differences in procedural success were observed across imaging subgroups.Abbreviations:IVUS, intravascular ultrasound; OCT, optical coherence tomography.

**Supplementary Table S7 Temporal imbalance and logistic regression sensitivity analyses by procedural period**

Part A Pre-matching distribution of procedural periods

| Characteristic | CAG (N = 163) | IVI (N = 305) | P |
| --- | --- | --- | --- |
| 3-category procedural period |  |  | 0.028 |
| 2015–2018 | 66 (40.5%) | 87 (28.5%) |  |
| 2019–2021 | 48 (29.4%) | 101 (33.1%) |  |
| 2022–2024 | 49 (30.1%) | 117 (38.4%) |  |
| Dichotomized 2-category period |  |  | 0.011 |
| 2015–2019 | 86 (52.8%) | 122 (40.0%) |  |
| 2020–2024 | 77 (47.2%) | 183 (60.0%) |  |

Notes:Pre-matching patient distribution across 3-category and dichotomized procedural periods, demonstrating significant temporal imbalance between CAG and IVI groups (P<0.05).

Part B MACE odds ratios of IVI under different temporal adjustment strategies

| Regression model | OR | 95% CI | P |
| --- | --- | --- | --- |
| Unadjusted model | 0.58 | 0.33–1.03 | 0.062 |
| Adjusted for 3-category period (primary) | 0.49 | 0.27–0.89 | 0.018 |
| Adjusted for 2-category period (sensitivity) | 0.51 | 0.29–0.93 | 0.026 |

Notes:Logistic regression sensitivity analyses evaluating the association between IVI guidance and MACE under different temporal adjustment strategies. MACE was treated as a binary outcome without accounting for time-to-event in these models.

**Supplementary Figure**

**Supplementary Figure S1 Diagnostic plots for the primary propensity score matching (PSM) cohort**

Figure legend:Legend(A) ：Density distribution of propensity scores in the unmatched overall cohort before matching; clear separation between the two groups indicates baseline imbalance that necessitated PSM.

Figure legend:Legend((B) Love plot showing absolute standardized mean differences (SMDs) of all covariates before (orange dots, unadjusted cohort) and after (blue dots, primary matched cohort) PSM. The vertical dashed line at SMD = 0.1 represents the threshold for acceptable baseline balance. Most covariates achieved SMD < 0.1 after matching; only dyslipidemia presented mild residual imbalance, which was fully resolved in the stringent expanded PSM sensitivity cohort (*Supplementary Figure S2*). Angio, CAG-only guidance group; IVI, intravascular imaging guidance group.

**Supplementary Figure S2 Covariate balance love plot for the stringent expanded propensity score matching (PSM) sensitivity cohort**

Figure legend: Absolute standardized mean differences (SMDs) of all baseline covariates before (red dots) and after (cyan dots) stringent expanded PSM. The vertical dashed line marks the SMD threshold of 0.1 for acceptable balance. All covariates achieved SMD < 0.1 after matching in this sensitivity cohort, eliminating residual baseline imbalance.

**Supplementary Figure S3 Kaplan-Meier survival curves for 12-month major adverse cardiac events (MACE) in the stringent expanded PSM sensitivity cohort**

Figure legend: Kaplan-Meier curves comparing cumulative MACE incidence between coronary angiography-guided (CAG, red line) and intravascular imaging-guided (IVI, cyan line) rotational atherectomy in the fully balanced sensitivity matched cohort. Log-rank test P = 0.045. The table below the curve displays the number of patients at risk at each follow-up time point (0, 3, 6, 9, 12 months).

**Supplementary Figure S4 LASSO variable selection and multicollinearity assessment for the propensity score-matched cohort**

Figure legend:
(A) Partial likelihood deviance curve from 10-fold cross-validation for LASSO penalized Cox regression. The vertical dashed line marks the optimal λ value minimizing partial likelihood deviance.

(B) LASSO coefficient profiles of all candidate predictors across varying log(λ) values. The vertical dashed line corresponds to the optimal λ selected via cross-validation, retaining three predictors with non-zero regression coefficients.

(C) Variance inflation factor (VIF) plot evaluating multicollinearity among covariates; all variables exhibited VIF < 5, indicating no substantial collinearity.

(D) Horizontal bar chart displaying regression coefficients of all variables with non-zero LASSO coefficients. To adhere to the events-per-variable (EPV) constraint and mitigate overfitting risk, only the two predictors with the largest absolute coefficients were retained in the final stratified Cox model, together with the study grouping variable (IVI/CAG guidance).

**Supplementary Figure S5 Temporal distribution of patients receiving CAG-only or IVI guidance before propensity score matching**

Figure legend:

(A) Bar plot displaying the number of patients receiving IVI-guided or CAG-guided rotational atherectomy across three discrete procedural periods (2015–2018, 2019–2021, 2022–2024).
(B) Bar plot showing patient counts stratified into two broad procedural eras (2015–2019, 2020–2024) according to intraprocedural imaging modality.
(C) Kernel density estimation curve reflecting the continuous temporal distribution of all enrolled patients in the IVI and CAG groups.

**Supplementary Figure S6 Comparison of rotational atherectomy procedural success rates by guidance strategy**

Figure legend:

(A) Bar chart displaying overall procedural success percentages in the CAG-only and IVI-guided groups. No significant intergroup difference was observed (χ² test, P=0.820).
(B) Stacked bar plot showing absolute counts of successful and failed rotational atherectomy procedures stratified by guidance modality.

**Supplementary Figure S7 Cumulative hazard curves of MACE stratified by procedural period**

Figure legend:

Cumulative hazard curves derived from stratified Cox regression stratified by three procedural periods (2015–2018, 2019–2021, 2022–2024). Curves show progressively higher long-term MACE risk in patients treated in later procedural eras, which violated the proportional hazards assumption and justified stratification by procedure period in the primary multivariable Cox model.
